# Bioinformatics analysis and population sample validation of ferroptosis-related genes in coronary heart disease: A case-control study

**DOI:** 10.1097/MD.0000000000047287

**Published:** 2026-01-23

**Authors:** Li-Heng Li, Cui-Hua Chen, Qing-Hua Dong, Yu-Bo Xie

**Affiliations:** a Department of Anesthesiology, The First Affiliated Hospital of Guangxi Medical University, Nanning, People’s Republic of China; b Intensive Care Unit, The Guilin Municipal Hospital of Traditional Chinese Medicine, Guangxi, China; c Department of Anesthesiology, Sichuan Clinical Research Center for Cancer, Sichuan Cancer Hospital and Institute, Sichuan Cancer Center, University of Electronic Science and Technology of China, Chengdu, China.

**Keywords:** bioinformatics analysis, coronary heart disease, G6PD, GLS2, NOX4, PRKAA2

## Abstract

The aim of this study was to investigate the association between coronary heart disease (CHD) and ferroptosis, furthermore, to identify the relevant genes and potential diagnostic biomarkers by bioinformatics analysis. We analyzed the CHD dataset GSE23561 from the gene expression omnibus database (http://www.ncbi.nlm.nih.gov/geo) and iron-death-related genes from the FerrDb database (http://www.zhounan.org/ferrdb/). By intersecting bioinformatics-identified differentially expressed genes with iron-death-related genes in FerrDb, we identified CHD associated genes linked to ferroptosis. These genes underwent gene ontology, Kyoto encyclopedia of genes and genomes pathway enrichment, and disease ontology analyses. Using LASSO logistic regression, we selected diagnostic biomarkers for coronary artery disease from these ferroptosis-related genes. Meta-analysis validated these biomarkers, followed by population sample validation recruited from our institution. This study has shown that genes associated with ferroptosis include PRKAA2, NOX4, GLS2, and G6PD. Further data analysis and meta-analysis revealed that NOX4 gene expression was significantly upregulated in patients with CHD (*P*<.05), which may be related to coronary atherosclerosis and myocardial injury. Additionally, the upregulation of NOX4 expression was validated through RT-PCR. The study identified 122 ferroptosis-associated genes, emphasizing their roles in oxidative stress, cellular membrane functions, and cardiovascular diseases. LASSO regression pinpointed 4 diagnostic biomarkers: PRKAA2, NOX4, GLS2, and G6PD, notably with elevated NOX4 expression in CHD patients. Meta-analysis and experimental validation confirmed NOX4’s significance in CHD, providing insights into its mechanisms and implications for early diagnosis and treatment strategies.

## 1. Introduction

Coronary heart disease (CHD) refers to the heart disease caused by coronary atherosclerosis under the action of a variety of pathogenic factors, which further leads to stenosis or complete occlusion of the coronary artery lumen, and ultimately leads to myocardial ischemia, damage or even necrosis. CHD is currently the major cause of the increasing incidence and mortality rate of cardiovascular diseases worldwide, and it is also the disease with the highest mortality rate globally.^[1]^ According to the data in the “Report on Cardiovascular Health and Diseases in China 2023,” the number of patients with cardiovascular diseases in China is estimated to be 330 million, with 11.39 million suffering from CHD.^[2]^ As people’s lifestyle changes, the overall patients with cardiovascular disease, diabetes, hypertension, hyperlipidemia tend to be younger. The patients suffering from CHD nowadays are becoming younger and no longer limited to the elderly. The number of acute myocardial infarction patients under the age of 35 is increasing with years.^[3]^ As a result, early diagnosis and treatment of CHD has become an urgent priority.

Cell death plays an important role in the normal development of cells and in maintaining homeostasis in the body, and it can also prevent excessive proliferation of various diseases. In recent years, researchers have discovered that autophagy, paraptosis, pyroptosis, oncosis, and other forms of cell death are also involved in different pathophysiological conditions. In 2012, Dixon et al.^[4]^ Proposed a novel form of cell death known as ferroptosis, which is iron-dependent. Ferroptosis is driven by the loss of activity of glutathione peroxidase 4 (GPX4) as a lipid repair enzyme and the accumulation of iron-dependent lipid reactive oxygen species (ROS), leading to cell death.^[5]^ This mode of cell death is involved in various pathological and physiological processes in the human body, including tumor development, neurodegeneration, and ischemia/reperfusion injury.^[6,7]^

The occurrence and development of CHD are mostly related to vascular endothelial dysfunction and atherosclerosis.^[8]^ The accumulation of oxidative metabolites in vascular endothelial cells (VECs) combined with oxidative stress leads to endothelial dysfunction. At the same time, the accumulation of lipid peroxide in cells is also an important mechanism for the occurrence of ferroptosis.^[9]^ Therefore, exploring the relationship between CHD and ferroptosis is of great significance for the pathogenesis research and prevention of CHD. The occurrence and progression of CHD are closely related to vascular endothelial dysfunction and atherosclerosis. The accumulation of oxidative metabolites in VECs, coupled with oxidative stress, leads to endothelial dysfunction. Additionally, the accumulation of lipid peroxides within cells is also an important mechanism for ferroptosis.^[10,11]^ Therefore, exploring the relationship between CHD and ferroptosis holds significant implications for understanding the pathogenesis of CHD and for exploring the corresponding prevention and treatment strategies.

## 2. Materials and methods

### 2.1. Data acquisition for coronary heart disease and ferroptosis-related genes

The coronary heart disease dataset (GSE23561) was downloaded from the gene expression omnibus (GEO) database (http://www.ncbi.nlm.nih.gov/geo). This dataset was selected through a systematic search using MeSH terms: “coronary artery disease (CAD),” “gene expression profiling,” and “humans.” The full dataset contains 35 samples across 5 groups: 9 normal controls, 6 rheumatoid arthritis samples, 6 metabolic syndrome samples, 6 CAD samples, and 8 type 2 diabetes samples. For this study, we specifically analyzed 9 normal control samples (GSM577963-GSM577971) and 6 CAD samples (GSM577984-GSM577989) as the target groups. A total of 259 ferroptosis-related genes were obtained from the FerrDb database (http://www.zhounan.org/ferrdb/legacy/operations/download.html (version2020), Driver (108) + Suppressor (69) + Marker (111) = 288, which is larger than the Gene count (259), because of 28 multi-annotated genes as shown in the figure on the website).

### 2.2. Identification of differentially expressed genes

Differential analysis on the GSE23561 dataset has been performed by means of the “limma” package in R language. Genes with adjusted *P* <.05 and |log2FC| ≥0.5 were selected for further analysis. The resulting differentially expressed genes were intersected with the ferroptosis-related genes from the FerrDb database to obtain the differentially expressed genes related to ferroptosis.

### 2.3. Functional enrichment analysis

Gene Ontology analysis, Kyoto encyclopedia of genes and genomes pathway enrichment analysis, and disease ontology disease enrichment analysis on the ferroptosis-related differentially expressed genes selected through the “Cluster Profiler” package in R language has been performed. Enrichment with an adjusted *P* <.05 was considered significant. Additionally, gene set enrichment analysis was conducted on the gene expression matrix, with |NES| > 1 and FDR < 0.25 indicating significant enrichment.

### 2.4. Selection of diagnostic biomarkers

LASSO logistic regression was employed for feature selection to identify diagnostic biomarkers for CAD. LASSO regression was used to further screen the differentially expressed genes related to ferroptosis in order to obtain the optimal diagnostic gene biomarkers.

### 2.5. Meta-analysis to validate the optimal diagnostic gene biomarkers

The mRNA expression chips from the GEO database related to CHD has been screened by the following search strategies: (CHD) OR (CAD) OR (CHD). After the preliminary retrieval through the above-mentioned search strategy, further screening of chips that meet the requirements has been carried out based on the following inclusion and exclusion criteria. The inclusion criteria are as follows: research subjects are human being; the dataset includes peripheral blood samples from both CAD patients and normal controls; the dataset contains mRNA expression data. Chips were excluded if they met any of the following criteria: research subjects are animals or plants; the samples in the dataset are not peripheral blood; the control group is absent in the dataset; the dataset does not contain mRNA expression data.

### 2.6. Validation of optimal diagnostic gene biomarkers in human

Samples of CHD patients (n = 32) and healthy controls (n = 26) were collected from The First Affiliated Hospital of Guangxi Medical University between July and August 2023. RT-qPCR analysis has been performed on peripheral blood leukocytes samples from both of the CHD patients and the healthy control group to verify the expression of optimal diagnostic gene biomarkers in the patients and healthy people. The primer sequence of NOX4 is 5’– CCCATCTGGTGAATGCCCTC −3’ (forward) and 5’– GAGGAATAGCACCACCACCA −3’ (reverse).

## 3. Results

### 3.1. Identification of ferroptosis-related differentially expressed genes

**Figure 1.**
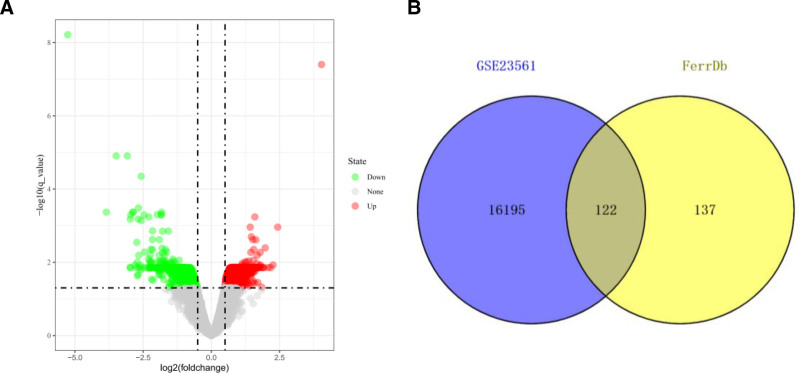
Shows the intersection between differentially expressed genes identified in the GSE23561 dataset (A) and ferroptosis-related genes provided by the FerrDb website (B) to obtain ferroptosis-related differentially expressed genes.

After screening with | log2 (FC) |>0.5 and corrected *P* <.05 as threshold value, 16,317 differentially expressed genes were identified in the GSE23561 dataset (Fig. 1A). And 122 related genes were obtained (Fig. 1B) when intersected with the 259 ferroptosis-related genes provided by FerrDb website, including 112 up-regulated genes. and 10 down-regulated genes.

### 3.2. Analysis of ferroptosis-related differentially expressed genes

Gene ontology analysis of the selected 122 genes indicates that biological processes was enriched in response to oxidative stress, cellular response to oxidative stress, ROS metabolic process; cellular components was enriched in: Caveola, membrane raft, membrane microdomain, membrane region; molecular function was enriched in: Cofactor binding, oxidoreductase activity acting on NAD(P)H, antioxidant activity.

**Figure 2.**
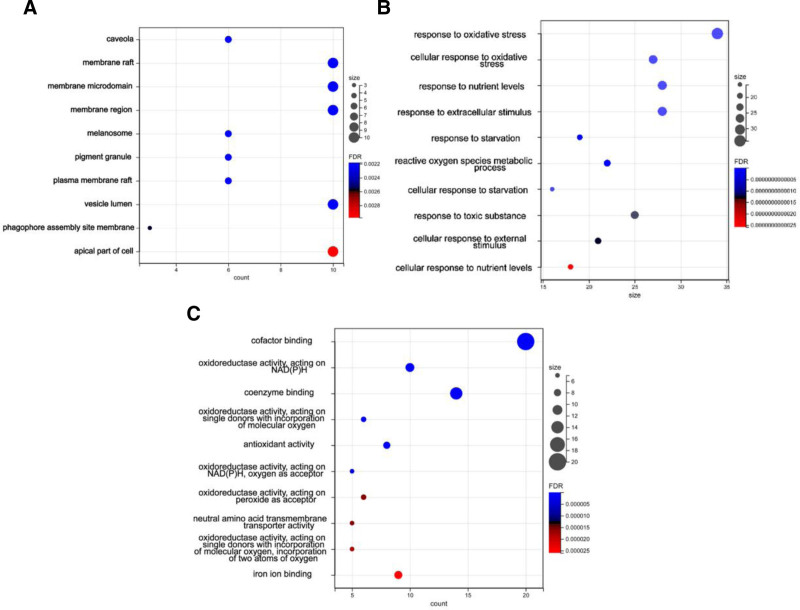
The filtered genes were subjected to GO analysis for ferroptosis-related differentially expressed genes. The results showed that the enriched results of BP, CC, and MF were mostly associated with oxidative stress and cell membrane functions. BP = biological processes, CC = cellular components, GO = gene ontology, MF = molecular functions.

The enrichment results reveal that the differentially expressed genes enrichment is mostly associated with oxidative stress and cellular membrane functions (Fig. 2).

**Figure 3.**
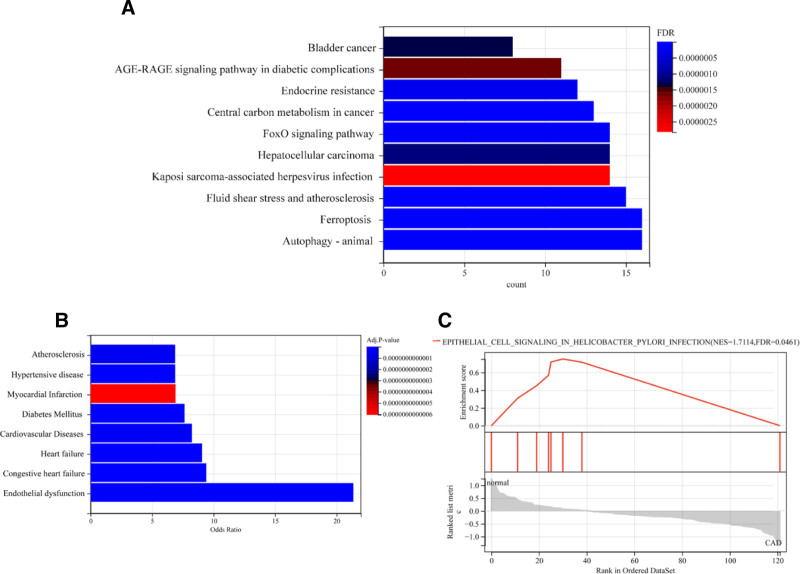
The filtered genes were subjected to KEGG pathway analysis. The results showed that the enriched genes were associated with ferroptosis, autophagy, and atherosclerosis (A). These genes were also found to be enriched in cardiovascular-related diseases in the disease enrichment analysis (B). Furthermore, conducting GSEA analysis on the differentially expressed genes revealed enrichment of pathways related to abnormal proliferation and motility of gastric epithelial cells (C). KEGG = Kyoto encyclopedia of genes and genomes.

Additionally, Kyoto encyclopedia of genes and genomes pathway analysis showed that genes were significantly enriched in pathways such as Ferroptosis, Autophagy–animal, and Fluid shear stress and atherosclerosis, indicating their relevance to ferroptosis, autophagy, and atherosclerosis, etc (Fig. 3A).

In terms of disease enrichment analysis, the differentially expressed genes were mainly associated with cardiovascular-related diseases such as heart failure and atherosclerosis (Fig. 3B).

The final gene set enrichment analysis analysis revealed that the differentially expressed genes were enriched in the EPITHELIAL_CELL_SIGNALING_IN_HELICOBACTER_PYLORI_INFECTION pathway, which is related to abnormal proliferation and motility of gastric epithelial cells (Fig. 3C).

### 3.3. LASSO regression identified 4 diagnostic gene biomarkers

**Figure 4.**
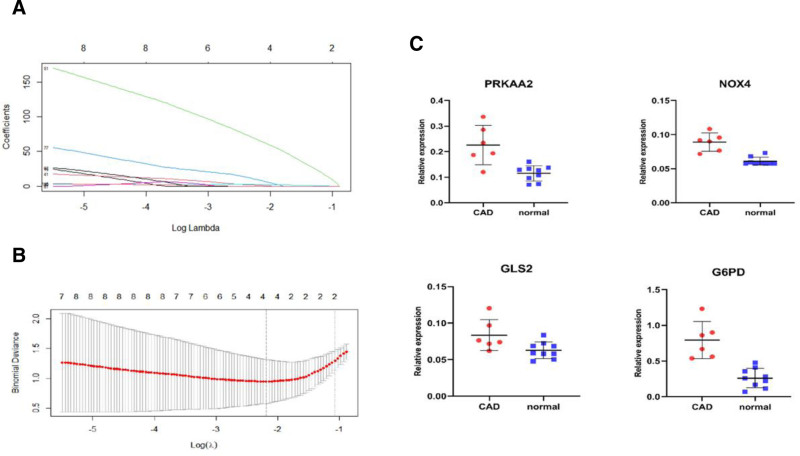
Presents the LASSO regression analysis of the screened ferroptosis-related differentially expressed genes, resulting in the identification of 4 hub genes with diagnostic significance: PRKAA2, NOX4, GLS2, and G6PD (A and B). These 4 genes were found to be highly expressed in the GSE23561 dataset (C).

Using LASSO regression, the 122 differentially expressed genes related to ferroptosis were further screened to identify hub genes with diagnostic value. Finally, 4 hub genes, PRKAA2, NOX4, GLS2, and G6PD, were obtained (Fig. 4A and B), all of which were highly expressed genes in the GSE23561 dataset (Fig. 4C).

### 3.4. Meta-analysis to validate the hub genes

**Figure 5.**
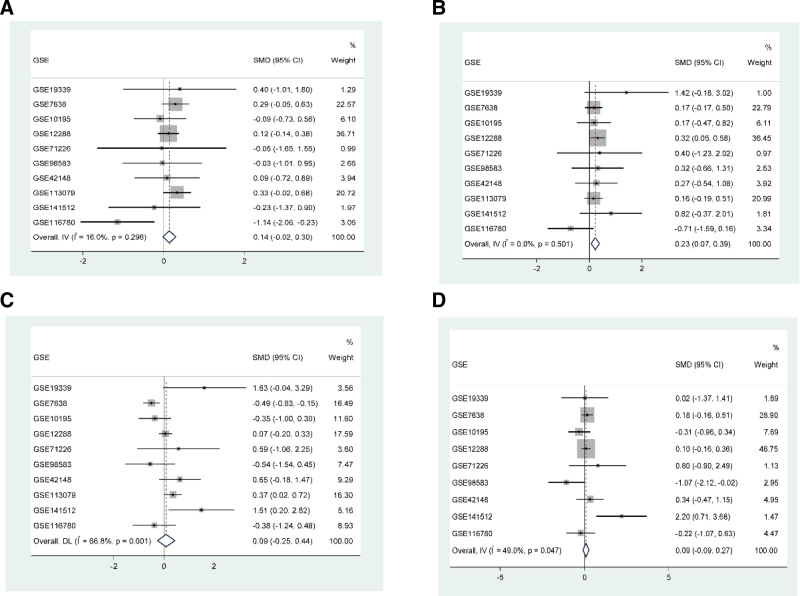
Displays the meta-analysis of the 4 hub genes in 9 datasets. Compared to the healthy control group, NOX4 mRNA was upregulated in the peripheral blood of CAD patients, while the analysis results for PRKAA2, GLS2, and G6PD were not significant. CAD = coronary artery disease.

A total of 9 GEO gene expression microarray datasets related to CAD, meeting the inclusion criteria, consist of 334 peripheral blood samples from CAD patients and 322 peripheral blood samples from healthy controls. These datasets are presented in Tables 1–4. Meta-analysis was conducted on 4 hub genes from the 9 datasets (Note: GSE113079 had no GLS2 expression). The results showed that compared to the healthy control group, the expression of NOX4 mRNA in peripheral blood of CAD patients was upregulated (SMD = 0.23, 95% CI [0.07–0.39], *P* = .006) (Fig. 5). However, the analysis results of PRKAA2, GLS2, and G6PD did not show any significant differences.

**Table 1:** PRKAA2 each data set expresses quantitative information.

GSE	CAD			normal		
	n	mean	SD	n	mean	SD
GSE19339	4	2.1657	0.12495	4	2.1299	0.02739
GSE7638	50	2.992	0.15843	110	2.9456	0.16122
GSE10195	27	0.1668	0.80828	14	0.2493	1.22126
GSE12288	110	5.1926	1.28815	112	5.0445	1.18077
GSE71226	3	5.3008	0.31987	3	5.3346	0.96107
GSE98583	12	3.1022	1.42037	6	3.1527	2.04328
GSE42148	13	2.1379	1.02522	11	2.0445	1.0968
GSE113079	93	−3.9523	0.62795	48	−4.1724	0.72976
GSE141512	6	2.4688	0.03681	6	2.48	0.05751
GSE116780	16	0.0417	0.05904	8	0.1409	0.127

CAD = coronary artery disease, SD = standard deviation.

**Table 2:** NOX4 each data set expresses quantitative information.

GSE	CAD			Normal		
	n	Mean	SD	n	Mean	SD
GSE19339	4	3.5491	0.89247	4	2.6531	0.05778
GSE7638	50	3.0191	0.1733	110	2.9914	0.15894
GSE10195	27	0.2037	0.84346	14	0.0345	1.18206
GSE12288	110	4.828	1.33173	112	4.4027	1.36514
GSE71226	3	3.4164	1.91929	3	2.7965	1.10774
GSE98583	12	3.9576	1.81005	6	3.4197	1.25568
GSE42148	13	2.8125	0.54666	11	2.6874	0.3446
GSE113079	93	−5.1054	0.5736	48	−5.21	0.78833
GSE141512	6	2.6522	0.02254	6	2.6295	0.03189
GSE116780	16	0.0264	0.04971	8	0.2002	0.42499

CAD = coronary artery disease, SD = standard deviation.

**Table 3:** GLS2 each data set expresses quantitative information.

GSE	CAD			Normal		
	n	Mean	SD	n	Mean	SD
GSE19339	4	3.491	0.15863	4	3.4876	0.1875
GSE7638	50	3.618	0.20385	110	3.5847	0.18285
GSE10195	27	−0.071	0.41787	14	0.074	0.56759
GSE12288	110	5.5236	1.0239	112	5.4225	0.97815
GSE71226	3	3.7228	0.12005	3	3.5688	0.24613
GSE98583	12	4.081	0.97612	6	5.0682	0.78488
GSE42148	13	6.1188	0.89089	11	5.8288	0.80692
GSE141512	6	3.9323	0.03157	6	3.8734	0.02104
GSE116780	16	0.029	0.05056	8	0.0391	0.03436

CAD = coronary artery disease, SD = standard deviation.

**Table 4:** G6PDEach data set expresses quantitative information.

GSE	CAD			Normal		
	n	Mean	SD	n	Mean	SD
GSE19339	4	6.4296	0.16766	4	6.1288	0.20048
GSE7638	50	7.6654	0.34974	110	7.808	0.26003
GSE10195	27	−0.1131	0.57804	14	0.0767	0.46659
GSE12288	110	8.2038	0.60216	112	8.1661	0.51571
GSE71226	3	6.1558	0.98515	3	5.7223	0.30659
GSE98583	12	9.3034	0.63633	6	9.602	0.2673
GSE42148	13	8.9515	0.61616	11	8.5939	0.46314
GSE113079	93	1.5278	0.29244	48	1.4201	0.29383
GSE141512	6	7.9133	0.12377	6	7.6737	0.18776
GSE116780	16	30.9216	19.22429	8	37.192	7.88142

CAD = coronary artery disease, SD = standard deviation.

### 3.5. Validation of NOX4 expression levels using RT-qPCR

**Figure 6.**
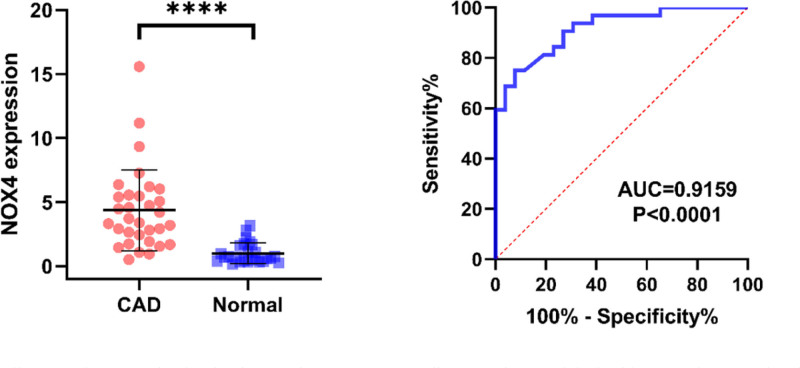
Illustrates the expression levels of NOX4 in coronary artery disease patients and the healthy control group using the RT-qPCR method. Independent sample t-tests were conducted to analyze the expression differences.

The expression levels of NOX4 for both of the samples of CAD patients and healthy control group were measured by RT-qPCR. An independent samples t test was performed to analyze their expression differences (Fig. 6). The results demonstrated that NOX4 expression was significantly up-regulated in CAD patients compared to the healthy control group, with a statistically significant difference (*P* <.0001).

## 4. Discussion

CAD is the most prevalent type of heart disease and one of the common causes of mortality of cardiovascular diseases worldwide which affects millions of people around the world. The mortality rate of it has shown a decreasing trend in recent years, however, it still ranks among the top in terms of mortality rates.^[12]^

Coronary atherosclerosis is a complex and progressive inflammatory disease, which is characterized by stenosis or occlusion of blood vessels caused by coronary artery lesions, resulting in myocardial ischemia, hypoxia and necrosis. CAD manifests across a spectrum of clinical symptoms, ranging from asymptomatic cases to stable angina, acute coronary syndrome, sudden cardiac death, or heart failure (HF).^[13,14]^ The progression of CAD is influenced by dysfunction of endothelial cells, smooth muscle cells, and macrophages, leading to the accumulation of atherosclerotic plaques.^[15]^ There may be individual differences among patients, some patients may experience sudden cardiac death due to acute coronary syndrome. The clinical symptoms of these patients who have developed clinical symptoms and sought medical attention timely can be alleviated through medication, percutaneous coronary intervention, or coronary artery bypass grafting surgery. However, due to challenges in early diagnosis, some patients might remain asymptomatic for years or even decades. Once detected, their prognosis is often unfavorable. Therefore, it is of great significance to have a deeper understanding of the intricate pathogenesis behind CAD and atherosclerosis.

Atherosclerosis is the basic pathogenesis of CHD. Aberrant apoptosis of VECs, macrophages, or vascular smooth muscle cells (VSMCs) is a shared characteristic of atherosclerosis, which may lead to the formation of atherosclerotic plaque or plaque instability. The repair of the damaged endothelial cells involves numerous genes, some of which also play roles in maintaining normal cardiac function and the formation of aortic aneurysms. Recent studies highlight ferroptosis as an iron-dependent form of cell death, primarily driven by iron overload-induced lipid peroxidation.^[4]^ David Carrick et al research indicates that increased myocardial iron content is associated with adverse left ventricular remodeling post myocardial infarction, and ferroptosis-induced oxidative stress is a significant contributor to CAD pathogenesis.^[16,17]^ Excessive intracellular free iron can generate abundant ROS through the Fenton reaction, accelerating the oxidation of low-density lipoprotein (LDL). Oxidized LDL is engulfed by macrophages, forming foam cells that deposit in vascular endothelium, expediting CAD progression.^[18]^ Lipid peroxidation in cell membranes forms the basis of cell ferroptosis.^[19]^ Bioinformatics analysis indicates NOX4’s involvement in oxidative stress-related pathways, closely related to cell membrane function. This suggests that NOX4 may promote lipid peroxidation, inducing ferroptosis and ultimately leading to lipid peroxidation damage, endothelial dysfunction, and CAD.

Beyond its established link to oxidative stress and ferroptosis, NOX4 exhibits distinct physiological roles that underscore its context-dependent significance in cardiovascular homeostasis. Under normal physiological conditions, NOX4, a major NADPH oxidase isoform in VECs and VSMCs, generates low levels of hydrogen peroxide to regulate essential processes: it participates in maintaining vascular tone by modulating endothelial nitric oxide (NO) signaling, supports angiogenesis through redox-dependent activation of pro-angiogenic pathways, and contributes to VSMCs differentiation and vascular remodeling during tissue repair.^[20,21]^ However, in the pathological milieu of CAD, sustained upregulation of NOX4 – triggered by pro-atherosclerotic stimuli such as angiotensin II, oxidized LDL, and chronic inflammation – tips the redox balance toward excessive ROS production.^[22]^ This dysregulated NOX4 activity amplifies lipid peroxidation beyond physiological thresholds, accelerates endothelial dysfunction by impairing NO bioavailability and promoting endothelial cell senescence, and drives VSMCs phenotypic switching (from contractile to synthetic phenotype), thereby facilitating plaque formation and instability.^[23,24]^ Clinically, elevated NOX4 expression in atherosclerotic plaques and circulating monocytes has been correlated with CAD severity, plaque vulnerability, and adverse cardiovascular outcomes, reinforcing its role as a key mediator bridging oxidative stress, ferroptosis, and CAD progression.^[25,26]^ These findings collectively highlight NOX4 as a dual-function molecule: its physiological redox signaling is vital for vascular health, but its pathological overactivation represents a critical therapeutic target in CAD.

In order to reduce error rate and make the results more reliable, a suitable gene chip and multiple microarray data sets and clinical samples were selected and studied through gene expression data analysis and bioinformatics enrichment methods. The result provides valuable clinical insights for CAD treatment and prevention. Furthermore, our bioinformatics analysis identified 4 ferroptosis-associated genes, namely PRKAA2, NOX4, GLS2, and G6PD. These genes are closely related to CAD and may also be involved in CAD patients’ cardiac immune microenvironment regulation. While gene expression may not be directly equivalent to protein expression, this study holds significant implications for understanding CAD mechanisms and potential therapeutic interventions.

## 5. Conclusion

Through bioinformatics analysis and population sample validation, this study has provided an in-depth exploration of ferroptosis-related genes in CHD. The results revealed 122 genes associated with ferroptosis, highlighting their significance in oxidative stress, cellular membrane functions, and cardiovascular diseases. LASSO regression identified 4 diagnostic biomarkers, namely PRKAA2, NOX4, GLS2, and G6PD, with NOX4 gene showing significantly elevated expression in CHD patients. Meta-analysis and experimental validation further validate the role of NOX4 in CHD. These findings offer new insights into the mechanisms of CHD as well as its early diagnosis and treatment strategies. Moreover, these findings will provide a valuable guidance for future research and clinical applications.

## Author contributions

**Conceptualization:** Yu-Bo Xie.

**Data curation:** Li-Heng Li, Cui-Hua Chen, Qing-Hua Dong.

**Formal analysis:** Li-Heng Li.

**Funding acquisition:** Yu-Bo Xie.

**Methodology:** Li-Heng Li, Yu-Bo Xie.

**Project administration:** Yu-Bo Xie.

**Software:** Li-Heng Li.

**Validation:** Li-Heng Li, Cui-Hua Chen.

**Writing – original draft:** Li-Heng Li.

**Writing – review & editing:** Li-Heng Li, Cui-Hua Chen, Qing-Hua Dong, Yu-Bo Xie.
